# Breed-associated risks for developing canine lymphoma differ among countries: an European canine lymphoma network study

**DOI:** 10.1186/s12917-018-1557-2

**Published:** 2018-08-06

**Authors:** Stefano Comazzi, Stefano Marelli, Marzia Cozzi, Rita Rizzi, Riccardo Finotello, Joaquim Henriques, Josep Pastor, Frederique Ponce, Carla Rohrer-Bley, Barbara C. Rütgen, Erik Teske

**Affiliations:** 1 Department of Veterinary Medicine, University of Milan, Via Celoria 10, 20133 Milan, Italy; 2 Department of Small Animal Clinical Science, Institute of Veterinary Science, University of Liverpool, Liverpool, UK; 3 Hospital Veterinario Berna, Lisbon, Portugal; 4 Department of Animal Medicine and Surgery, University Autonoma of Barcelona, Barcelona, Spain; 5 Department of Internal Medicine, University of Lyon, Lyon, France; 6 Division of Radiation Oncology, Department of Small Animals, Vetsuisse Faculty, University of Zurich, Zurich, Switzerland; 7 Department of Pathobiology, University of Veterinary Medicine, Vienna, Austria; 8 Department of Clinical Sciences of Companion Animals, Faculty of Veterinary Medicine, Utrecht University, Utrecht, Netherlands

**Keywords:** Dog, Lymphoma, Breed risk, Predisposition, Odds ratio

## Abstract

**Background:**

Canine breeds may be considered good animal models for the study of genetic predisposition to cancer, as they represent genetic clusters. From epidemiologic and case collection studies it emerges that some breeds are more likely to develop lymphoma or specific subtypes of lymphoma but available data are variable and geographically inconsistent. This study was born in the context of the European Canine Lymphoma Network with the aim of investigating the breed prevalence of canine lymphoma in different European countries and of investigating possible breed risk of lymphoma overall and/or different lymphoma subtypes.

**Results:**

A total of 1529 canine nodal lymphoma cases and 55,529 control cases from 8 European countries/institutions were retrospectively collected. Odds ratios for lymphoma varied among different countries but Doberman, Rottweiler, boxer and Bernese mountain dogs showed a significant predisposition to lymphoma. In particular, boxers tended to develop T-cell lymphomas (either high- or low-grade) while Rottweilers had a high prevalence of B-cell lymphomas. Labradors were not predisposed to lymphoma overall but tended to develop mainly high-grade T-cell lymphomas. In contrast with previous studies outside of Europe, the European golden retriever population did not show any possible predisposition to lymphoma overall or to specific subtypes such as T-zone lymphoma.

**Conclusion:**

Further prospective studies with more precise and consistent subtype identification are needed to confirm our retrospective results and to create the basis for the investigation of possible genes involved in different predispositions.

**Electronic supplementary material:**

The online version of this article (10.1186/s12917-018-1557-2) contains supplementary material, which is available to authorized users.

## Background

The study of cancer epidemiology in canine populations has earned a focus of attention, because tumour bearing dogs may help in better understanding a broad spectrum of determinants that may contribute to cancer development. In contrast to human populations, canine breeds can be considered as genetic clusters. Hence, they may provide genetic population information for studies on incompletely understood genetic disease predisposition [1, 2]. In addition, the great similarities of the disease complex, similar incidence and shared risk factors further support the use of canine lymphoma as a model for the human counterparts. To date, few specific studies on the epidemiology of canine lymphoma compared to the whole canine population have been published [3–11] (Additional file 1: Table S1).

Further data on the prevalence of specific lymphoma subtypes among different breeds can be found in larger lymphoma studies [12–25] (Additional file 2: Table S2). From these data taken together, it emerges that lymphoma is over-represented in the Doberman, Bernese mountain dog, Rottweiler, boxer, and bullmastiff breeds, independently of geographic origin. However, for other breeds, and particularly for the golden retriever, the high prevalence of lymphoma is found solely outwith Europe. Specific studies comparing European (EU) and extra-EU caseloads are currently lacking.

In addition to lymphoma in general, differing predispositions to lymphoma subtypes and immunophenotypes have been identified among canine breeds [25]. Interestingly, a high prevalence of golden retriever was documented in non-EU dogs with T-zone lymphoma [13, 17, 21, 24] but EU studies have not reached the same conclusion [7, 15].

When all data are analysed together, the risk of lymphoma overall and the distribution of specific lymphoma subtypes varies among different countries with golden retrievers experiencing even greater inconsistencies in distribution than other breeds.

The present retrospective study was designed in the context of the European Canine Lymphoma Network, a community of researchers working on lymphoma diagnosis and cure, which now incorporates more than 80 researchers from throughout Europe. Our aim was to investigate the breed prevalence in lymphoma in clinical caseloads from multiple European institutions. Data were compared to adequate control groups to define the overall breed risk of lymphoma in general, or in a specific lymphoma subtype.

## Methods

### Inclusion criteria

Databases of 8 different institutions based in different European countries (Austria, France, Italy, Netherlands, Portugal, Spain, Switzerland and United Kingdom) were retrospectively searched for consecutive cases of canine nodal lymphoma within a six year timeframe (2010–2015). Institutions enrolled were all referral centers for veterinary oncology, all located in academia, except one private referral center. Criteria for inclusion included a final diagnosis of lymphoma, based on results of clinical and laboratory data (variably including cytology, immunophenotyping or histopathology, depending on institution) and the availability of signalment data. As a control, the same institution provided a list of consecutive canine cases, from the same hospital databases with a diagnosis other than lymphoma, in the same timeframe. These control groups were used to compare the prevalence for each breed in the lymphoma group, in order to correct for other possible sources of variation, such as high breed popularity in one area. The number of control cases varied among institutions but in all cases they exceeded the number of lymphoma cases (generally at least 10 fold higher). Data from dogs with lymphoma were analysed after matching with the appropriate control from the same geographic area in order to correct results for the breed prevalence among the whole canine population.

When immunophenotype and lymphoma subtype were available these data were also recorded. Since diagnostic algorithms and classification schemes differ greatly among institutions, the subtypes recognized were grouped into 3 main groups: 1) B-cell lymphoma (all subtypes), 2) T-cell lymphoma-high grade, 3) T-zone lymphoma/T indolent lymphoma (identification based on histopathology, cytology or immunophenotyping).

### Statistical analysis

The 9 most represented breeds in the whole lymphoma database were considered for statistical purposes. The prevalence of different breeds in the databases from different countries varied but these 9 selected breeds were always among the 12 most common breeds for each country included in the study. We limited the analysis to these breeds in order to make comparisons between different institutions consistent.

Using country-matched control groups obtained from internal databases of each institution, odds ratio (OR) analysis was performed to quantify the association, if any, between lymphoma predisposition and breed within each country. ORs were expressed with relative 95% confidence interval.

The differences between control and lymphoma groups were evaluated using Chi-square test.

A breed was considered predisposed if *p* < 0.05 and OR > 1.0. When OR > 3.0 (corresponding to a moderate positive risk) a breed was defined as highly predisposed, as used previously in Ernst et al. [5].

Multinomial logistic regression was used to define the predisposition to a specific lymphoma subtype in the 9 chosen breeds (in comparison with the crossbred population), with lymphoma subtype the response variable and breed the explanatory variable. B-cell lymphoma was considered as reference of the response variable; therefore, two models were constructed for each breed: a model comparing high-grade T-cell to B-cell lymphoma and a model comparing T-zone to B-cell lymphoma. Results are reported as odds ratios (OR) with the associated 95% confidence intervals (CI).

Statistical analysis was performed using PROC LOGISTIC (SAS 9.4, SAS Inst. Inc., Cary, NC).

### Ethics

The University of Liverpool ethics committee approved the study (number VREC442). According to national regulations and to the Animal Experimentation Ordinance of the “Comitato Etico Scientifico per la Sperimentazione Animale” of the University of Milan (EC decision 29 October 2012, renewed with the protocol n° 02–2016), the other institutions involved in this international research project did not require further permissions to proceed with this retrospective study, as data had already been acquired during normal clinical activity.

## Results

A total of 1529 canine lymphoma cases and 55,529 control cases were collected among all institutions.

The following 9 breeds were over-represented among lymphoma-affected dogs: Labrador retriever (*n* = 91, 5.5%), boxer (*n* = 88; 5.1%), German shepherd (*n* = 72; 4.2%), golden retriever (*n* = 71; 4.1%), Rottweiler (*n* = 62; 3,6%), Bernese mountain dog (*n* = 60; 3.5%), Doberman (*n* = 35; 2.0%), English cocker spaniel (*n* = 33; 1.9%) and beagle (*n* = 28; 1.6%).

**Fig. 1:**
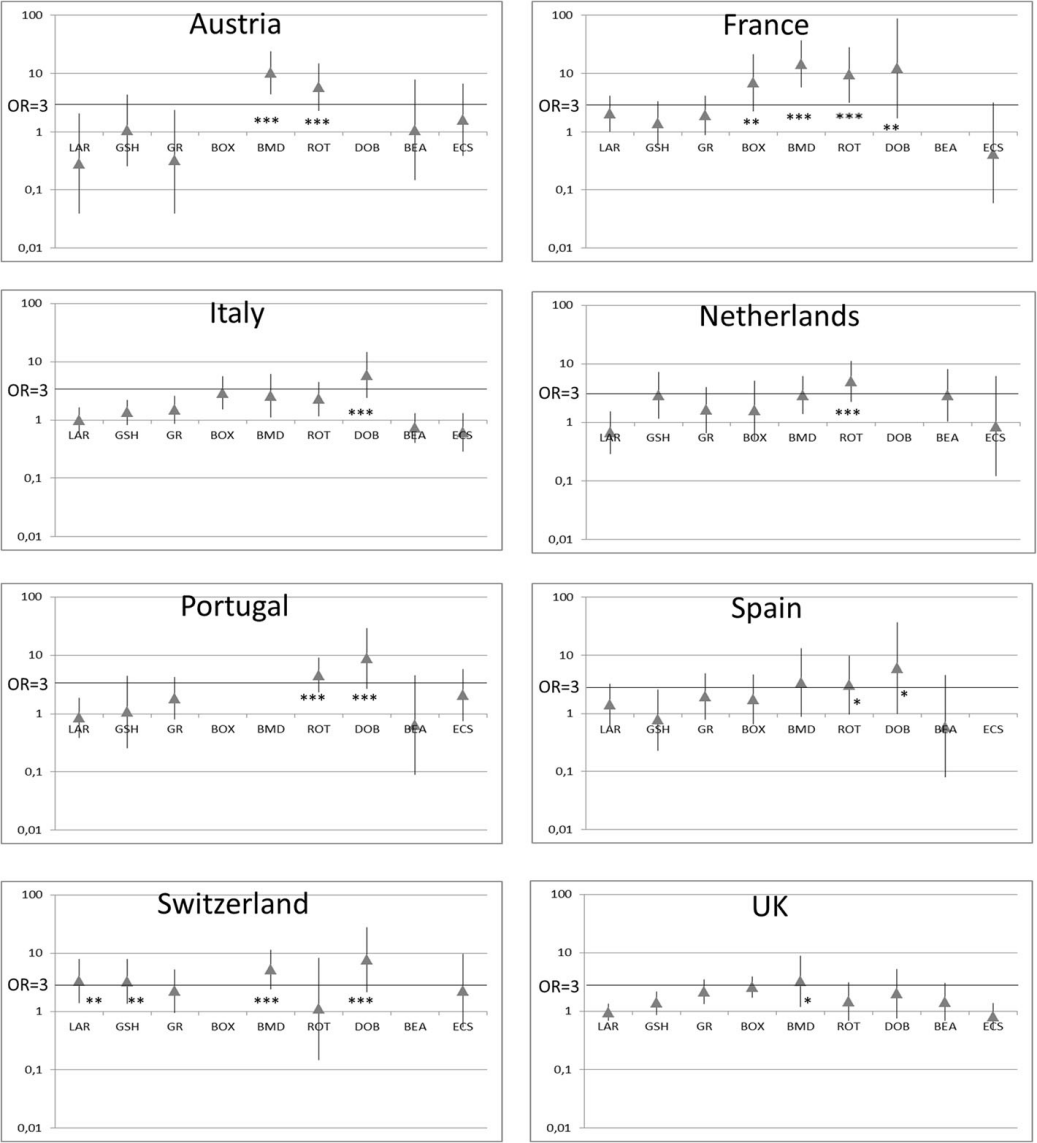
Odds Ratio (OR) and confidence interval to develop lymphoma for each canine breed in each country. *P* values are expressed for OR > 3 suggesting a high predisposition. LAR = Labrador Retriever, GSH = German Shepherd; GR = Golden Retriever; BOX = Boxer; BMD = Bernese Mountain Dog; ROT = Rottweiler; DOB = Doberman; BEA = Beagle; ECS = English Cocker Spaniel; ns = not significant;*p < 0.05; **p < 0.01; ***p < 0.001

Prevalence varies among different countries, partly reflecting the prevalence of the breeds in their local canine population. OR for lymphoma were calculated for each breed in single countries and are shown in Fig. 1 and Table 1. Briefly, Rottweilers and Dobermans showed a statistical predisposition to develop lymphoma (OR > 3) in 5 out of 8 countries, Bernese mountain dog in 4 out of 8; while German shepherd and Labrador retriever were found to be predisposed just in Switzerland and boxer just in France. Golden retrievers were found to be mildly predisposed just in the UK (OR = 2.16; confidence limit = 1.34–3.49) while no predisposition was recorded in other countries.

**Table 1:** Odds Ratio (OR) values for lymphoma that results significant to statistical analysis

	Portugal	Italy	United Kingdom	Spain	Austria	Switzerland	France	Netherlands
	OR	OR	OR	OR	OR	OR	OR	OR
Labrador Retriever	ns	ns	ns	ns	ns	3.33**	2.05*	ns
German Shepherd	ns	ns	ns	ns	ns	3.27**	ns	2.9*
Golden Retriever	ns	ns	2.16*	ns	ns	ns	ns	ns
Boxer	ns	2.92***	2.56***	ns	ns	ns	6.89**	ns
Bernese	ns	2.6*	3.25*	ns	10.18***	5.26***	14.39***	2.92**
Rottweiler	4.59***	2.29*	ns	3.06*	5.78***	ns	9.39***	4.99***
Doberman	8.84***	5.89***	ns	6.05*	ns	7.66***	12.15**	ns
Beagle	ns	ns	ns	ns	ns	ns	ns	ns
English Cocker Spaniel	ns	ns	ns	ns	ns	ns	ns	ns

OR Values higher that 3 are in bold

*ns* not significant

*p < 0.05; **p < 0.01; ***p < 0.001

For analysis of lymphoma subtypes, all countries were grouped together as the number of cases having a lymphoma subtype classification was too low to perform analysis by countries. Immunophenotype and cyto-histotype data were available in 1446 dogs and were used to categorize cases into the above mentioned main groups: B-cell lymphoma (*n* = 950; 65,7%), T-cell lymphoma-high grade (*n* = 396; 27,4%), and T-zone lymphoma/T indolent lymphoma (*n* = 100; 6,9%). No statistical differences were found in the prevalence of lymphoma subtypes between the whole canine population and crossbreeds. Results of the prevalence of lymphoma subtypes compared with crossbreeds used as controls are shown in Table 2.

**Table 2:** different lymphoma subtypes no (percentage in brackets) in different breeds and Odds Ratio (OR) values (with confidence interval) in comparison with crossbreeds (XXX)

	B-cell lymphoma		HG T-cell lymphoma		T-zone lymphoma		TOT
		OR vs XXX		OR vs XXX		OR vs XXX	
Labrador Retriever	44 (53.0%)	ref	35 (42.2%)	**2.74 (1.61–4.67)**	4 (4.8%)	1.40 (0.44–4.49)	83
German Shepherd	52 (80%)	ref	11 (16.9)	0.73 (0.36–1.49)	2 (3.1)	0.59 (0.13–2.70)	65
Golden Retriever	48 (71.6%)	ref	16 (23.9)	1.15 (0.61–2.17)	3 (4.5%)	0.96 (0.26–3.51)	67
Boxer	12 (21.4%)	ref	38 (67.9%)	**10.9 (5.36–22.3)**	6 (10.7%)	**7.69 (2.49–23.8)**	56
Bernese Mountain Dog	41 (78.9%)	ref	10 (19.2%)	0.84 (0.40–1.78)	1 (1.9%)	0.38 (0.05–2.95)	52
Rottweiler	56 (94.9%)	ref	2 (3.4%)	**0.12 (0.03–0.52)**	1 (1.7%)	0.28 (0.03–2.15)	59
Dobermann	30 (85.7%)	ref	4 (11.4%)	0.46 (0.16–1.36)	1 (2.9%)	0.51 (0.07–4.06)	35
Beagle	18 (66.7%)	ref	7 (25.9%)	1.34 (0.53–3.37)	2 (7.4%)	1.71 (0.36–8.18)	27
English Cocker Spaniel	18 (60%)	ref	11 (36.7%)	2.11 (0.94–4.71)	1 (3.3)	0.86 (0–11-6.91)	30
Crossbreeds	200 (73.8%)	–	58 (21.4%)	–	13 (4.8%)	–	271

Significant data are in bold

*Ref* Reference value

From our results, it emerges that boxer and Labrador retriever were predisposed to develop high-grade T-cell lymphomas (respectively 10.9 fold and 2.7 fold compared with crossbreeds) while the Rottweiler was highly predisposed to have B-cell lymphoma (8.1 fold more likely than high-grade T-cell lymphoma). Boxers also showed a predisposition to T-zone lymphoma in comparison with B-cell lymphoma but no difference was found when compared with high-grade T-cell lymphoma. Interestingly, no predisposition to T-zone lymphomas was found in golden retriever in comparison to either B-cell lymphomas or high-grade T-cell lymphomas.

## Discussion

Reported canine breed predispositions to lymphoma and different lymphoma subtypes are scarce and often inconsistent among studies. Dog breeds, as genetic clusters, represent an excellent model to study genetic predisposition to oncogenesis. In our study, the breed prevalence of lymphoma was investigated in clinical caseloads from 8 European countries/institutions, and was compared to adequate control groups in order to define breed risks of developing lymphoma overall and of developing different lymphoma subtypes. In order to quantify our findings and minimize possible biases due to control group or any statistical artifacts, we arbitrarily defined a breed as highly predisposed when OR was higher than 3 (moderate to high risk).

Results from the present study confirm the prior suggested predisposition of Bernese mountain dogs, Rottweilers, and Dobermans of developing lymphoma. These results are in agreement with previous findings of breed predispositions both in- and outside Europe [3, 8].

Other breeds showed a wider variation among different countries.

Boxers were found to be highly predisposed only in France (OR = 6.89) although a mild to moderate statistical predisposition (OR lower than the value of 3) was also found in Italy (OR = 2.92) and the UK (OR = 2.56). In addition, this result supports a previous report from France [7], in which Boxers were the only breed found to be predisposed to lymphoma in that country. The same predisposition was confirmed in other published studies from Australia [3], USA [8] and UK [9] . In summary, boxers could be considered as a breed at-risk of developing lymphoma in Europe.

Conflicting results arise from analysis of the golden retriever population, which only showed a modest predisposition for developing lymphoma in the UK (OR < 3). Golden retrievers have however been reported to be highly predisposed to lymphoma development in a study from the USA [8]. This breed was generally over-represented in American and Japanese canine lymphoma case series, often constituting more than 20% of cases [13, 17, 21, 24] . Recently, geographic variation between different regions of the USA in the prevalence of T-zone lymphoma in American golden retriever has been reported [12]. This regions varying prevalence among lymphoma subtypes was suggested to be a possible consequence of environmental risk factors, as already demonstrated in humans [26].

Environmental risk factors may explain the different breed risks observed between American and European studies, but also possible is differing genetic predisposition to lymphoma between American and European golden retrievers breed lineages. The two breed lineages are particularly different in terms of morphotype, with minimal interbreeding. Existing genetic differentiation between “American” and “European-British” golden retrievers have been detected in research on mast cell tumors [27]. Although we consider risk factors as an important source of bias in our cases, we think the genetic explanation as more likely, as no differences were observed in term of predisposition to lymphoma in general and/or T-zone lymphoma between European countries (regardless of non-homogeneous distribution of possible risk factors). Assessing the role of environmental risk factors was beyond the scope of the present study, but we expect they might influence the percentage of lymphoma cases in a certain area irrespective of breed. In contrast, we found no difference in the prevalence of lymphoma in the whole canine population or in mixed breeds among European and American lymphoma case series. The effects and interaction between environmental risk factors and breed-related predisposition would be better investigated in a prospective study collecting information related to environment, exposure to pollutants and owners’ habits.

Breed prevalence of T- vs B-cell lymphoma has been evaluated by several authors. Modiano et al. [25] found a higher prevalence of T-cell lymphoma in boxers and golden retriever; and B-cell lymphoma in Dobermans in comparison with crossbreed dogs. More recently a predilection for T-cell lymphoma in boxers was demonstrated in a Polish study [6]; this was also confirmed by Lurie et al. [28], with a predilection for the T lymphoblastic subtype (high-grade T-cell lymphoma) in boxer dogs. Unfortunately, the Polish study did not differentiate between high- and low-grade T- cell lymphoma, a distinction that is important not only in terms of prognosis and clinical approach but also in terms of possible genetic predisposition.

In our study, we aimed to differentiate the lymphoma subtypes in the investigated population, when possible. However, since classification schemes, diagnostic pathways and nomenclature varied markedly between institutions, we elected to separate the lymphoma cases in 3 different classes: B-cell lymphoma, high-grade T-cell lymphoma, and low-grade T-cell/T-zone lymphoma. These divisions allowed collection of an adequate number of cases in each class, with separation of the differing prognoses between classes. We decided not to separate high-grade and low-grade B cell lymphomas due to: 1) the high prevalence of high-grade lymphoma vs low-grade in dogs; 2) the difficulty in defining the grade in some B-cell lymphoma subtypes (such as marginal lymphoma, late stage); 3) the paucity of studies on different outcome in nodal high vs low-grade B-cell lymphomas. Indeed, a median lymphoma-specific survival of 13 months was found in marginal zone lymphoma (the most diffuse low-grade B-cell lymphoma), which did not differ from survival reported in high-grade B-cell lymphoma [21].

Our results confirm the already reported predisposition of boxers for T-cell lymphoma in general but they also underline the possible predispositions of Labrador retriever to high-grade T-cell lymphoma and of Rottweilers to B-cell lymphomas, which have not previously been described. The absence of a more precise classification of lymphoma subtype (Peripheral T Cell vs lymphoblastic for T cell lymphomas, Diffuse Large B Cell vs marginal vs follicular for B cell lymphomas) does not permit further conclusion to be drawn on these possible predispositions, but opens the door to more specific studies with a consistent classification. One of the most exciting results of our findings is that in Europe golden retrievers do not have a higher probability of developing T-zone lymphoma, either compared to B-cell lymphoma or high-grade T-cell lymphoma in its own right. This finding differs from the majority of studies published in the USA and Japan and opens new perspectives on different genetic predispositions to lymphomagenesis, considering that golden retriever populations differ in terms of morphology and share minimal interbreeding. Confirmation of our results with a prospective study using a standardized classification method would be useful to clarify this finding, before focusing on identification of genetic regions involved in the different predispositions.

Limitations of the present study should be considered and are mainly secondary to its retrospective nature. Firstly, a common standard classification for all cases was not available, since classification schemes (WHO vs updated Kiel classification), diagnostic algorithms and techniques used varied among different institutions. In order to provide consistency between data, collected cases were categorized where possible into three categories (B cell lymphoma, irrespective from the grade; high-grade T cell lymphoma and T zone lymphoma). Although incomplete, this categorization allowed to provide more information than the simple diagnosis of lymphoma and to include the majority of cases. However, in many cases, subtype identification was not provided or it was based just on an incomplete panel of tests (e.g. cytology alone). These cases could not be subdivided into the three categories and were excluded from statistical analysis on subtypes. The lack of a common classification in different subtypes could affect the meaning of our results regarding possible breed predispositions to B cell lymphomas and T high grade lymphomas. However, it could be considered more valid for T zone lymphomas, as they represent a more homogenous lymphoma subtype with well- recognized biological behaviour. More specific studies could be performed by using a standard diagnostic approach (including cytology, histopathology, and immunophenotyping in all cases) and a common unique classification scheme in order to confirm the data derived from this retrospective analysis and to better identify breed-specific predilections that could be then used for possible genetic studies.

The second limit is due to the choice of control cases. Non-lymphoma cases were selected from the same database used for the extraction of lymphoma cases, in order to correct results for the breed distribution, which is highly variable among countries. These databases included diseased dogs presented at clinical examination or tissue samples sent for laboratory tests (cytology, flow cytometry). The breed prevalence in these databases is not necessarily representative of the whole canine population in each country. However, we considered it the most reliable control group available, since breed data on overall canine populations were not available for all countries, and are not consistent between European countries and areas. Bias could occur as all the institutions derive their database solely from diseased animals, and breed distribution may vary according to different breed predisposition to other diseases. However, due to the high number of control cases and the variety of possible diseases included, we considered this as a minor bias for the breed analyzed.

## Conclusions

The results of the present study confirm that in spite of some differences among countries, some dog breeds are predisposed to developing lymphoma in general and some predisposed to specific canine lymphoma subtypes. This is particularly true for the Rottweiler, suffering a higher prevalence of B-cell lymphoma and the boxer, with a higher prevalence of T-cell lymphoma (either high-grade or T-zone lymphoma). Other breeds such as the Doberman and Bernese Mountain dog are highly predisposed to the development of lymphoma in general with no specific subtype predisposition. In contrast, the golden retriever, previously reported as predisposed to the development of lymphoma and particularly to T-zone lymphoma in the USA, did not show any particular predisposition in the herein investigated European case series.

## Additional files

Additional file 1: **Table S1.** Breed prevalence in literature: summary of previous studies on breed prevalence for canine lymphoma in different countries. (DOCX 15 kb)

Additional file 2: **Table S2.** Lymphoma subtypes in golden retriever: summary of previously published papers on prevalence of different lymphoma subtypes in golden retriever:, from different countries. (DOCX 15 kb)
