# Supplementary material for: Breed-associated risks for developing canine lymphoma differ among countries: an European canine lymphoma network study
Source: BMC Vet Res. 2018 Aug 6;14:232. doi: 10.1186/s12917-018-1557-2 (PMC6090884; doi:10.1186/s12917-018-1557-2)
Supplement: Supplementary file 1 — Table S1. Breed prevalence in literature: summary of previous studies on breed prevalence for canine lymphoma in different countries. (DOCX 15 kb) [file 12917_2018_1557_MOESM1_ESM.docx]

**Additional file 1**

**Table S1:** Summary of previous studies on breed prevalence for canine lymphoma in different countries.

| **Reference** | **Country** | **no of cases** | **Predisposed breeds** |
| --- | --- | --- | --- |
| Yau et al., 2017 ^3^ | New South Wales, Australia | 134 | **Doberman, Australian cattle dog, Rottweiler, Border collie, Boxer** |
| Gruntzig et al 2016 ^4^ | Switzerland | 2955 | Rottweiler, Swiss mountain dog |
| Ernst et al., 2016 ^5^ | Germany | 411 | American pitbull terrier, American staffordshire terrier, Briard, Irish setter, Bernese mountain dog. |
| Yankowska et al., 2015 ^6^ | Poland | 336 | **Briard, Bernese mountain dog, Dogue de Bourdeaux**, Boxer, Rottweiler |
| Pastor et al., 2009 ^7^ | France | 608 | **Boxer**, Setter, Cocker spaniel |
| Villamil et al., 2009 ^8^ | USA | 14573 | **Bullmastiff, Boxer, Bernese mountain dog, Scottish terrier, Gordon setter, Irish wolfhound, Basset hound, Golden retriever**, Rottweiler, others |
| Modiano et al. 2005 ^25^ | USA | 1263 | Golden retriever, Shi-tzu, Cocker spaniel, Siberian husky, Doberman, Boxer, Bassethound |
| Edwards et al., 2003 ^9^ | United Kingdom | 103 | **Bullmastiff, Bulldog, Boxer.** |
| Jagielski et al., 2002 ^10^ | Poland | 63 | **Rottweiler** |
| Teske, 1994 ^11^ | Netherlands | 254 | **Scottish terrier, Boxer,** Bouvier des Flandres, Rottweiler. |

*Breeds in which OR are >3 are written in bold.*
