# Supplementary material for: Breed-associated risks for developing canine lymphoma differ among countries: an European canine lymphoma network study
Source: BMC Vet Res. 2018 Aug 6;14:232. doi: 10.1186/s12917-018-1557-2 (PMC6090884; doi:10.1186/s12917-018-1557-2)
Supplement: Supplementary file 2 — Table S2. Lymphoma subtypes in golden retriever: summary of previously published papers on prevalence of different lymphoma subtypes in golden retriever:, from different countries. (DOCX 15 kb) [file 12917_2018_1557_MOESM2_ESM.docx]

**Additional file 2**

**Table S2:** Prevalence of different lymphoma subtypes in Golden Retriever: summary of previously published papers, from different countries.

| **Reference** | **Country** | **No of cases** | **Lymphoma subtypes** | **% Golden Retrievers** |
| --- | --- | --- | --- | --- |
| Gavazza et al, 2008 ^23^ | Italy | 120 | all lymphomas | <5% |
| Martini et al, 2015 ^15^ | Italy | 51 | T-zone lymphoma | <2% |
| Aresu et al., 2015 ^14^ | Italy | 63 | all lymphomas | 5% |
| Poggi et al., 2015 ^16^ | Italy | 90 | B-cell lymphoma | 1% |
| Marconato et al., 2013 ^19^ | Italy | 46 | B-cell lymphoma | 9% |
| Joetzke et al, 2012 ^22^ | Germany | 44 | all lymphomas | 5% |
| Seelig et al.,2014 ^17^ | USA | 494 | T-zone lymphoma | 40% |
| Valli et al., 2006 ^24^ | USA | 10 | Indolent lymphoma | 20% |
| Flood-Knapik et al., 2012 ^21^ | USA | 75 | Indolent lymphoma | 35% |
| Modiano et al., 2005 ^25^ | USA | 1263 | all lymphomas | 19% |
| Frantz et al., 2013 ^20^ | USA | 80 | all lymphomas | 63% |
| Graff et al., 2014 ^18^ | USA | 107 | B-cell lymphoma | 12% |
| Ruple et al, 2016 ^12^ | USA | >5000 | all lymphomas | 9% |
| Mizutani et al., 2016 ^13^ | Japan | 16 | T-zone lymphoma | 56% |
